# Palladium-Doped Tin Oxide Nanosensor for the Detection of the Air Pollutant Carbon Monoxide Gas

**DOI:** 10.3390/s20205889

**Published:** 2020-10-17

**Authors:** Jeyapaul Sam Jebakumar, Asokan Vimala Juliet

**Affiliations:** Department of Electronics and Instrumentation, SRM Institute of Science and Technology, Kattankulathur 603203, Tamil Nadu, India; jsjebakumar@gmail.com

**Keywords:** air pollution, CO, SnO_2_ nanoparticles, XRD, FESEM

## Abstract

The exhaust gases from various sources cause air pollution, which is a leading contributor to the global disease burden. Hence, it has become vital to monitor and control the increasing pollutants coming out of the various sources into the environment. This paper has designed and developed a sensor material to determine the amount of carbon monoxide (CO), which is one of the major primary air pollutants produced by human activity. Nanoparticle-based sensors have several benefits in sensitivity and specificity over sensors made from traditional materials. In this study, tin oxide (SnO_2_), which has greater sensitivity to the target gas, is selected as the sensing material which selectively senses only CO. Tin oxide nanoparticles have been synthesized from stannous chloride dihydrate chemical compound by chemical precipitation method. Palladium, at the concentration of 0.1%, 0.2%, and 0.3% by weight, was added to tin oxide and the results were compared. Synthesized samples were characterized by X-ray diffraction (XRD) and field emission scanning electron microscope (FESEM) techniques. XRD revealed the tetragonal structure of the SnO_2_ nanoparticles and FESEM analysis showed the size of the nanoparticles to be about 7–20 nm. Further, the real-time sensor testing was performed and the results proved that the tin oxide sensor, doped with 0.2% palladium, senses the CO gas more efficiently with greater sensitivity.

## 1. Introduction

Air pollution has become the fifth leading risk factor worldwide for mortality [1]. No one is safe from air pollution because the waste from many processes, technically termed as air pollutants, are dumped directly into the environment, which exhibits a direct impact on the environment and thereby endangering the natural ecosystem. The most abundant sources of air pollutants are gases, among which those of primary concern are oxides of carbon (CO_2_ and CO), SO_2_, and NO_x_ [2].

Carbon monoxide, an invisible air pollutant, is odorless, tasteless, and non-irritant, but is very toxic and can lead to damage of heart and the central nervous system, becoming lethal at times. Because of insufficient oxygen that leads to incomplete combustion in the operation of vehicles, heating, coal power generation, and biomass burning, CO is produced as a toxic gas [3]. In many countries, the most common type of fatal air poisoning is CO poisoning [4]. When the source of the toxic gas is at the surface of the earth and if it has residence time in the order of several days, this trace gas becomes a basic pollutant, like in the case of CO [5]. Owing to meteorological conditions and emission patterns, the emission of CO showed a seasonal, weekly, and diurnal cycle [6]. Myronuk (1977) [7], had reported long back that the exposure of humans to high concentrations of carbon monoxide can result in headache or nausea, the control of which when failed, leads to setting a strain on heart and lungs. Further studies have confirmed that breathing high concentrations of CO reduces the blood’s ability to carry oxygen by hemoglobin and also causes other health issues like headaches, nausea, vomiting, hypotension, weakness, inflammation of existing diseases, depression and hearing problems [8]. Even at low levels (~9 ppm for 8 h of respiration), CO is toxic to human beings and all life forms. Hence there is a significant need to measure CO levels by using a sensor that can be specific to CO gas even in the parts-per-million range.

In the design of a CO gas sensor, selection of an appropriate sensing material stays as the first and foremost step, which needs to address the type of sensor, the nature of target gas, and the concentration of the detective gas. Literature reviews show that the sensitivity, selectivity, and response speed of the gas-sensing properties are higher in the gas sensors based on nanomaterials [9]. Nanomaterials are well-known to possess excellent electrical, optical, thermal, catalytic properties, and strong mechanical strength, which offer great opportunities to construct nanomaterials-based sensors or devices for monitoring environmental contaminations in air, water, and soil [10]. These sensors have many advantages like small size and optical band gap, as well as increased surface area [11] and fast response [12], when compared to traditional sensors.

The advantage of metal oxide gas sensors, over other sensors, are their low cost, quick production, firm size, and easy measuring electronics, because of which they find their use in portable gas detection systems [13]. Furthermore, when metal oxide semiconductors are compared, tin oxide (SnO_2_) is a widely studied metal oxide semiconductor which shows greater sensitivity to the target gases [14] and is used to detect many gases in practical application. A comparison of gas-sensing properties among four different metal oxide thin films, namely, tungsten dioxide (WO_2_), tungsten trioxide (WO_3_), tin oxide (SnO_2_), and tin-doped tungsten trioxide (Sn-doped WO_3_), shows SnO_2_ to be the best thin film for sensing acetone gas [15]. Tin oxide is a good sensing material to find out the concentration of CO even at a high temperature [16] and therefore, in this study, SnO_2_ is selected as the sensing material.

In order to enhance the gas-sensing properties, SnO_2_ nanomaterials with different morphologies and spatial assemblies have been fabricated and reported [17]. Noble metals are effective oxidation catalysts and this property makes them useful to enhance the reactions on gas sensor surfaces. Doping noble metals with metal oxide nanocomposite structures enhances the sensitivity as well as the stability of the sensors and lowers the working temperature [18]. This clearly indicates that doping of noble metals like Au, Ni, Co, Cu, Pt, Pd, etc., enables to increase the sensitivity of the sensing material. The mesostructured Au-doped SnO_2_ had highly selective response toward CO [19] and Ni-doped SnO_2_ nanoparticles-based gas sensor exhibited better gas response than pure SnO_2_ [20]. When cobalt (Co)-doped multilayer SnO_2_ was tested toward carbon dioxide, it showed a high response of 41%, whereas undoped SnO_2_ showed a weak response [21]. On the other hand, when copper (Cu)-doped SnO_2_ films are used in sensing measurements, Cu-doping was found to improve the response of the tin oxide sensor to CO gas [22]. When SnO_2_ synthesized by hydrothermal method was doped with PdPt by in situ reduction of metallic salt and measured for its response to gaseous methane, the results demonstrated that the dopants can increase the response of SnO_2_ sensor toward methane and also improve the stability during continuous cycles [23]. Moreover, the Pd-SnO_2_ composite nanoporous structure showed prominent methane gas-sensing ability as compared with pure nanoparticles and this sensor also exhibited high repeatability and long-term stability [24]. Earlier it was shown that highly uniform SnO_2_ nanowires act as sensitive, fast, stable, and reproducible gas sensors, that can be easily integrated into a multi-component array and the gas-sensing can be enhanced when functionalized with palladium catalyst [25].

SnO_2_ is a semiconductor of the n-type, which has a wide band gap of about 3.6 eV at 300 K, and finds its application in catalysts, sensors, lithium battery electrodes, and electrochemical energy storage [2]. This, when heated in air at high temperature, oxygen gets adsorbed on the particle surface by capturing free electrons, leading to changes in electrical conductivity of SnO_2_ grains, that forms the working principle of SnO_2_ as gas sensors. SnO_2_ nanoparticles show higher sensitivity on gas sensors because of their large surface areas and thus, when the size of the particles is minimized, the performance of the sensors can be made better [26]. The sensitivity of SnO_2_, formed by the method of solid-state reaction, toward CO gas is maximal when worked at a temperature of 275–300 °C [27], though the working temperature range of SnO_2_-based gas sensor is from 25 °C to 500 °C. At low temperature, SnO_2_ also senses few more gases like CO_2_, HC, and NO_x_. Hence it is necessary to increase its selectivity even at low temperature. Palladium (Pd) has very good activity and outstanding sensitivity toward carbon monoxide at very low temperature [28].

With these background literatures, in this study, we have identified a sensing material SnO_2_ doped with palladium, which is capable of sensing CO gas among various other gases. The sensing material was then examined by X-ray diffraction for the structure of the particles and field emission scanning electron microscopy techniques for the structure morphology. In the development of Pd-doped SnO_2_-based hydrogen sensors, the best performances were observed for 0.5% Pd [29]. It has been identified very much earlier that the response of SnO_2_-based sensors lies in the range of 0.1–0.6% for noble metals [30]. In our study, the pollutant to be detected is carbon monoxide, which is heavier than hydrogen. When the response of the composite graphene-SnO_2_ is studied with 0.1%, 0.5%, and 1% Pd, 0.1% Pd-doped composite showed the highest response and was found to be the optimum Pd-doping concentration [31]. So, we designed this study to be performed with three concentrations of Pd ≥ 0.1%, viz., 0.1%, 0.2%, and 0.3%, along with stannous chloride dehydrate compound, and are taken as three different samples. The real-time testing of the sensor was also performed to verify its sensitivity and reliability.

## 2. Experimental Methods

### 2.1. Synthesis of SnO_2_

**Figure 1:**
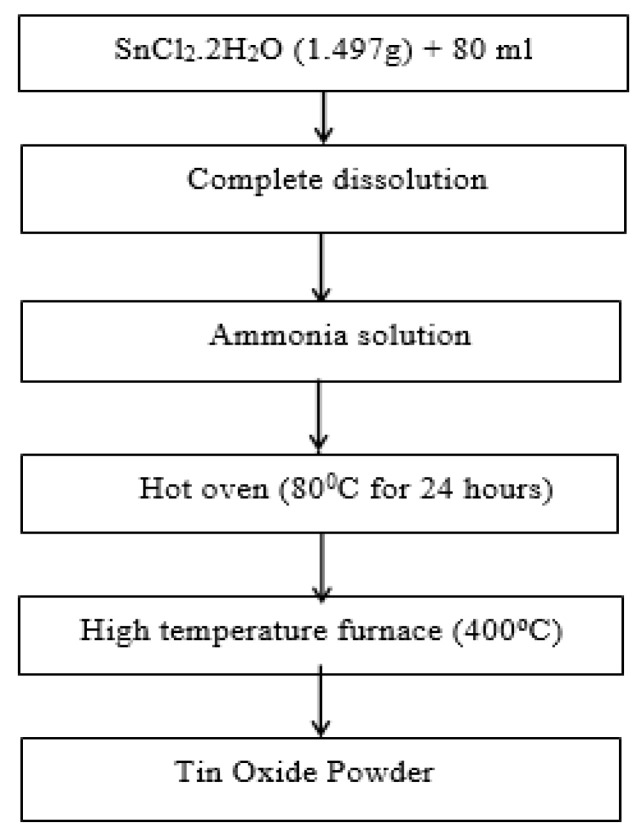
Synthesis process flow of SnO_2_.

SnO_2_ nanoparticles were synthesized by simple chemical precipitation synthesis using SnCl_2_.2H_2_O as an inorganic precursor. Stannous chloride dehydrate (SnCl_2_.2H_2_O) was dissolved in 80 mL distilled water in a beaker with continuous stirring. After complete dissolution, ammonia was added drop wise into the solution as a reagent to control the pH, when pure dense tin oxide is obtained [32]. The synthesis of SnO_2_ powder is shown as a flow chart in Figure 1.

The solution was stirred to homogeneity and the mixture was kept aside until the formation of gel substances. Once gel substances were formed, the excess water was filtered and the resulting gel was kept in hot oven at 80 °C for 24 h to remove water molecules from the gel. The dried samples were furnaced at 400 °C for 2 h [33]. To increase the sensitivity toward carbon monoxide, palladium was added with stannous chloride dehydrate. To investigate the sensitivity of the sample with different palladium concentrations, the same procedure was repeated by adding 0.1%, 0.2%, and 0.3% by weight of palladium to stannous chloride dehydrate in three different beakers. The crystalline powder obtained from all the four samples synthesized by chemical precipitation method, was carefully stored for further analysis.

### 2.2. X-ray Diffraction

X-ray diffraction method is the classic technique used to determine the crystal structure of any unknown material and hence, tin oxide crystalline powder synthesized by chemical precipitation method was characterized by X-ray diffraction (XRD; PANalytical, The Netherlands) having generator settings of 40 mA, 45 kV, and CuKα radiation with a wavelength λ =1.541 Å at 2θ values within 20° and 80°. X-ray detector rotates at an angle of 2θ and the data are collected at this angle, for typical powder patterns. The Bragg’s law conditions are contented by various d-spacings in crystalline materials, when the angle θ is varied. The angular degree and intensity are plotted in counts of the obtained diffraction peaks that produced a pattern which is distinctive of the sample. All the four samples synthesized are delicately ground, blended, and the mean particle size is determined. In the X-ray scan, the angles are fixed from 10° to 90° [34].

If the wavelength of X-ray is represented by *λ*, the full width at half maximum of the diffraction peak by *B*, and the Bragg diffraction angle by *θ*, the crystallite size (*D*) of the prepared powders can be determined using the Scherrer equation [35]

(1) $$D=\frac{0.9\lambda }{B\cos\theta }$$

Molecular mechanics and molecular dynamics simulations were combined with X-ray powder diffraction to investigate the structure [36].

### 2.3. Field Emission Scanning Electron Microscopy

Field emission scanning electron microscopy is used for investigating topographic information of specimens at extremely high magnifications of 40,000× to 800,000× with practically unlimited field depth. The morphologies of the samples were examined using field emission scanning electron microscopy (FESEM; FEI, Quanta 200, The Netherlands), and images were taken at the same magnification of 80,000×. The working distance was adjusted until a crisp focus was achieved, and this working distance was varied between 10 mm and 11 mm. In order to confirm the chemical composition of the samples, FESEM equipped with energy-dispersive X-ray (EDX) was used [37].

### 2.4. Real-Time Testing of the Sensor

**Figure 2:**
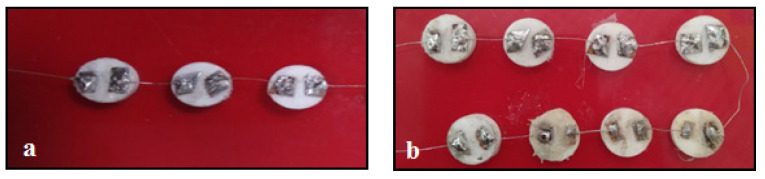
Contact pads fixed on alumina substrate with the coated sensing material connected by lead-coated copper wires in series of (**a**) three sensor array (**b**) eight sensor array.

The contact pads were fixed at 10-mm diameter alumina substrate and the sensing material was coated on it, as depicted in Figure 2. In order to compare the sensitivity, the alumina substrate with the coated sensing material, in three numbers (Figure 2a) and eight numbers (Figure 2b), was connected in series. Mostly, for bonding semiconductor devices, fine gold wires or doped or alloys of gold wires are used [38]. But because of the increased cost of the metal, copper wires coated with lead, was tried and it turned out to be effective for bonding.

**Figure 3:**
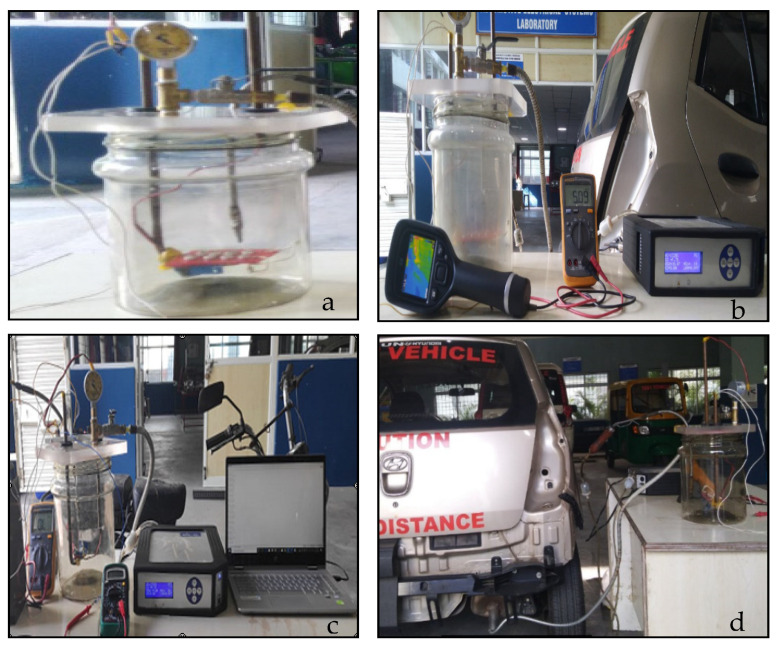
Experimental set-up for real-time sensor testing. (**a**) Sensor arrangement inside chamber with an exhaust gas analyser and a commercial CO sensor MQ-7. (**b**) Gas chamber with sensor arrangement connected to the measurement setup using external lead. (**c**) Data collection from chamber by the data acquisition system, continuously recording the values. (**d**) Test-vehicle engine connected to the inlet of the chamber.

To verify and validate the experiment, an exhaust gas analyser (AVL DIGAS 444N) and one commercial carbon monoxide gas sensor (MQ-7) were kept inside the gas chamber, the arrangement of which is shown in Figure 3a. Contact pads on the substrate, connected to lead-coated copper wires, were further connected to the measurement setup using external lead and this entire experimental set-up is shown in Figure 3b. The output of the gas sensors were connected in the analogue input ports of the data acquisition system (MYDAQ, NI USB-6009) and the values were continuously recorded, as shown in Figure 3c. The air inside the chamber was evacuated with the help of vacuum pump and once vacuum was created, the exhaust from petrol engine was connected to the inlet of the chamber, as in Figure 3d.

The engine was maintained at an ideal speed of 1000 rpm. The exhaust gas was permitted to go inside the chamber for 30 min (ON) to check the sensor response and the exhaust was stopped for 30 min by closing the inlet as well as opening the chamber outlet, to check the recovery time of the sensor. The carbon monoxide gas present in the exhaust mixture chemically reacts with SnO_2_ and the resistance of SnO_2_ changes. The potential difference of sensor array was recorded as a function of time and then the air was passed inside for 30 min to check the recovery of the sensor. The same experiment was repeated by coating the substrate with the four different sensing materials synthesized, connected in series as 3 sensor array and 8 sensor array, and the responses were recorded respectively.

## 3. Results and Discussion

The synthesized powder was examined by X-ray diffraction and field emission scanning electron microscopy techniques, the former indicated the structure of the particles and the latter investigated the structure morphology. The real-time sensor testing was then carried out to verify if CO gas could be sensed and the sensing properties were compared, which showed that the tin doped with 0.2% Pd exhibited better sensing ability.

### 3.1. Structural Properties of SnO_2_ by XRD Analysis

**Figure 4:**
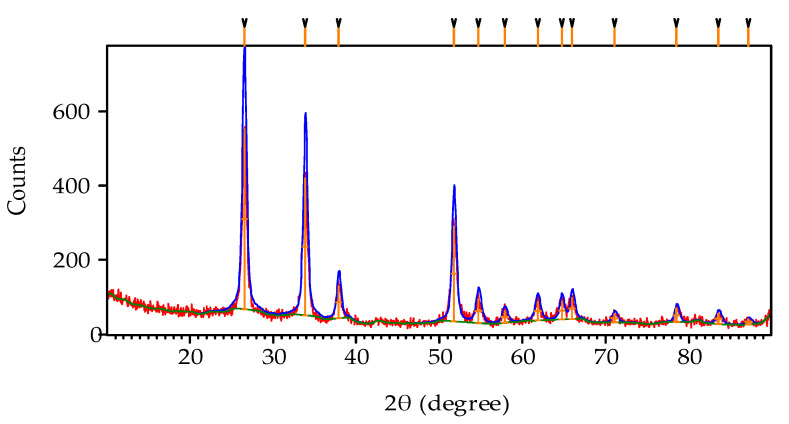
XRD pattern showing the peaks that matched the product to be SnO_2_.

The XRD pattern obtained by the SnO_2_ product is depicted in Figure 4. Pattern started from 10.03° and ended at 89.95° with continuous scan type at 25 °C. The peaks seen at 2θ values of 26.58°, 33.87°, 37.95°, 38.98°, 42.64°, 51.77°, 54.76°,61.88°, and 65.97° can be associated with (1 1 0), (1 0 1), (2 0 0), (111), (210), (2 1 1), (2 2 0), (3 1 0), (3 0 1) respectively [36]. The observed standard (hkl) planes, when matched, finalized that the product SnO_2_ is pure [39]. Having a structure with the literature values (JCPDS card no. 01-088-0287), (ICSD Collection code: 084576), and (ICSD NAME: Tin Oxide).

**Figure 5:**
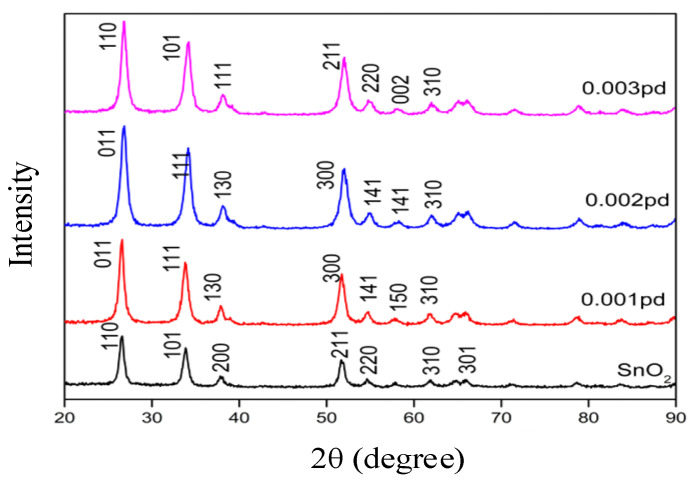
X-ray diffraction intensity of pure SnO_2_, SnO_2_ with 0.1% Pd (labeled as 0.001 pd), SnO_2_ with 0.2% Pd (labeled as 0.002 pd) and SnO_2_ with 0.3% Pd (labeled as 0.003 pd), given in count values at an angle 2θ.

Similarly, the remaining samples also have reached the peaks at 2θ and have matched standard (hkl) planes that are briefly explained as comparison in Figure 5, which also shows the lattice parameter values. All matched intensity peaks are noted with lattice parameter values. The experimental results obtained clearly depict the structure of the synthesized products, or in other words, the sensing material. When CO sensing mechanisms were investigated with first principles calculations, it was shown that CO molecule grabs oxygen from the pre-adsorbed O on the Pd_4_ n or PdO cluster on the tin oxide (110) surface, resulting in the formation of CO_2_ [40]. The sensitivity of tin oxide to CO or any target gas is recently shown to be triggered by oxygen bound to Sn on the nanosheet, because of the conversion of the valency state of tin by the adsorption and desorption of oxygen on the 101 surface compared to the 110 surface [41]. Palladium can replace Sn and thus cause more vacancies for oxygen to be adsorbed on the sensor surface, thereby improving the gas sensor performance [31].

From Figure 5, we can understand that the intensity counts of each sample differ because of its crystalline size and the percentage of palladium added to the SnO_2_ sample. When the SnO_2_ sensor is doped with 5 wt% Pd^2+^ ions, it exhibits higher electric conductance and gas sensing properties to characteristic fault hydrocarbons, with fast response and recovery, at 350–400 °C [42]. Developed NO_2_ sensors using 1 mol% Pd-loaded In_2_O_3_ hierarchical microstructures showed exceptional NO_2_ detection at a further lower operating temperature of 110 °C, though other prevalent chemicals like H_2,_ CO, CH_4_, and ethanol were present [43]. The performance of palladium nanoparticles compared to the bulk Pd is more because of the increased surface to volume ratio that further leads to a higher effective surface for the interaction of palladium [44]. Thus, addition of palladium to SnO_2_ nanoparticles will tend to increase the sensitivity toward carbon monoxide and the gas-sensing property.

### 3.2. Morphological Properties of SnO_2_ by FESEM Analysis

**Figure 6:**
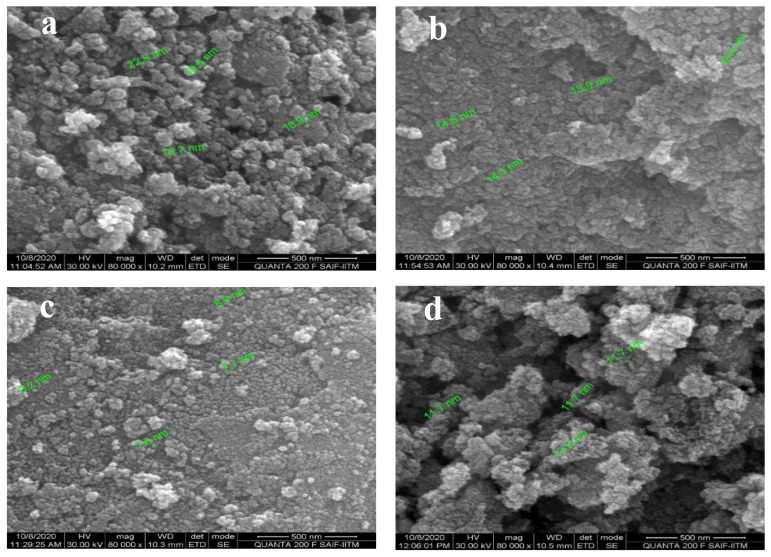
FESEM image showing the particle size of (**a**) SnO_2_, (**b**) SnO_2_ with 0.1% Pd, (**c**) SnO_2_ with 0.2% Pd, (**d**) SnO_2_ with 0.3% Pd.

Field emission scanning electron microscope was used to analyze the morphological properties of the synthesized SnO_2_ sample, and cluster of nanoparticles occurred on the surface. It is clear from the FESEM analysis that the SnO_2_ particles are crystalline in character and the results reveal their crystalline structure [45]. The result of FESEM analysis at magnification 80,000× is shown in Figure 6. In Figure 6a, it is seen that the average particle size for SnO_2_ is 20 nm. The result in Figure 6b, which is the FESEM analysis for SnO_2_ with 0.1% Pd, shows that the average particle size is 15 nm. FESEM analysis for SnO_2_ with 0.2% Pd, as seen in Figure 6c, shows that the average particle size is 8 nm. Similarly, from FESEM analysis for SnO_2_ with 0.3% Pd, as depicted in Figure 6d, it is observed that the average particles size is 12 nm. A careful examination of these structures reveals the presence of tin oxide nanoparticles of average size around 7–20 nm, the smallest particle size being observed for the sample containing SnO_2_ and 0.2% Pd. This spherical morphology with a greater surface area is perhaps the reason for the increased sensitivity [25].

**Figure 7:**
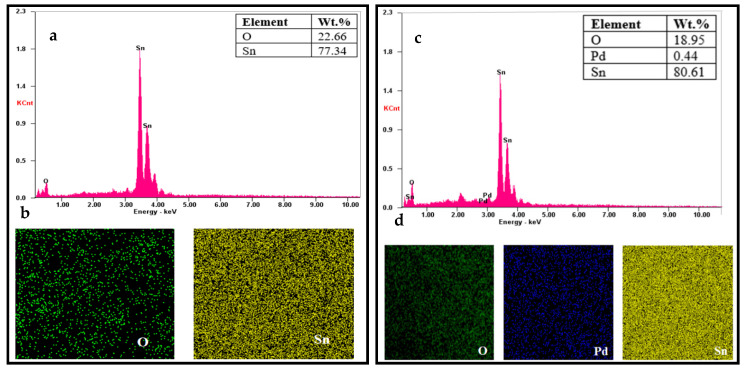

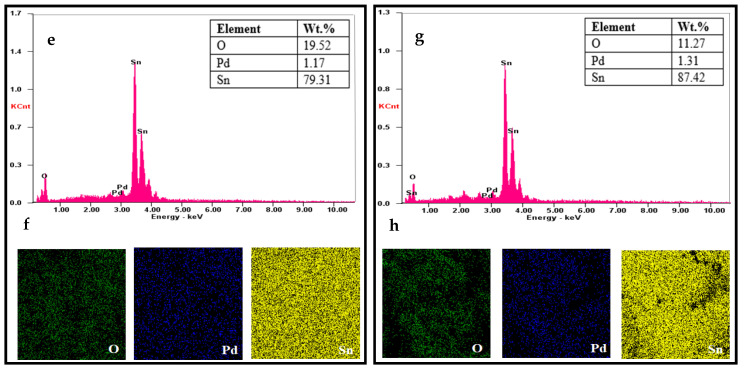
Energy-dispersive X-ray elemental mapping analysis. (**a**) EDX spectrum of SnO_2_. (**b**) EDX mapping of elements O and Sn in SnO_2_. (**c**) EDX spectrum of SnO_2_ with 0.1% Pd. (**d**) EDX mapping of elements O, Pd, and Sn in SnO_2_ with 0.1% Pd. (**e**) EDX spectrum of SnO_2_ with 0.2% Pd. (**f**) EDX mapping of elements O, Pd, and Sn in SnO_2_ with 0.2% Pd. (**g**) EDX spectrum of SnO_2_ with 0.3% Pd. (**h**) EDX mapping of elements O, Pd, and Sn in SnO_2_ with 0.3% Pd.

The EDX spectrum of SnO_2_ (Figure 7a) verified the presence of Sn and O, whereas that of SnO_2_ with 0.1% Pd (Figure 7c), SnO_2_ with 0.2% Pd (Figure 7e), and SnO_2_ with 0.3% Pd (Figure 7g) confirmed the presence of Sn, O, and Pd. Further, EDX mapping of SnO_2_ (Figure 7b), SnO_2_ with 0.1% Pd (Figure 7d), SnO_2_ with 0.2% Pd (Figure 7f), and SnO_2_ with 0.3% Pd (Figure 7h) illustrated extensive dispersion of Sn, O, and Pd elements, thereby confirming that they are well-distributed on the surface.

It is interesting to note that the size of the nanoparticles decreases drastically on adding 0.1% and 0.2% palladium, however the addition of 0.3% Pd, increases the size of the nanoparticles. The nanostructures of the SnO_2_ nanoparticles are strongly influenced by doping that can be attributed to the surface to volume ratio, thereby enhancing the agglomeration and leading to increase in particle size. When a sensor was developed with nano-sized PdO loaded on SnO_2_ nanoparticles, the maximum of the sensor response was obtained in 0.1 mol% PdO and the particle size of PdO increased with loading amount because of the agglomeration of PdO nanoparticles to each other [46]. Likewise, above a particular concentration of the dopants Ni and Co, the radii of the doped ZnO increased the crystallite size resulting in a significant increase of the particle size [47]. Palladium enhancing the sensing capability of SnO_2_ is attributed to the presence of Pd clusters on the surface of tin oxide support, as well as the incorporation or embedding of Pd into the support [48]. It has been proved that palladium remains in its oxidized state and finely dispersed, when added in small quantities (0.2 wt%), whereas, it forms clusters at higher concentrations [49]. Thus, the increase in size of the nanoparticles on adding 0.3% Pd, may be due to the formation of clusters or the agglomeration of the nanoparticles, after attaining a level of saturation. As the particle size is found to influence the gas-sensing property closely, the increase in size of the nanoparticles will tend to decrease the CO sensing ability of 0.3% Pd.

### 3.3. Real-Time Testing of the Sensor

**Figure 8:**
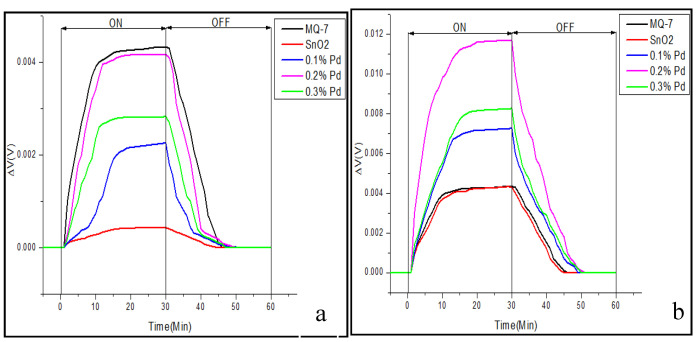
Comparison of change in voltage for pure SnO_2_ as well as with different concentrations 0.1%, 0.2%, 0.3% of palladium and commercial CO sensor MQ-7, tested for 60 min @1000 rpm, connected as (**a**) three sensor array and (**b**) eight sensor array.

The three main characterization parameters for the performance of any sensor are sensitivity, response time, and recovery time. Response time is the time for responding to a step concentration change from 0 to 90% of the saturated value and recovery time is the time taken for the signal of the sensor to return to 90% of the initial value [50]. From the results in Figure 8a,b, it is observed that the eight sensor array coated with the sensing material of SnO_2_ and 0.2% Pd, gives 11.6 mV, compared to 4.1 mV in three sensor array, which is because of the additive effect of the potential. The accepted mechanism in the detection of carbon monoxide, a reducing gas, is the partial chemical reduction of the surface of the sensitive SnO_2_ layer of nanoparticles, thereby leading to an increase in its conductivity, which can be increased by using a sensor array [51]. Our results clearly show that increasing the sensor array number from three to eight has significantly increased the sensitivity. In the eight sensor array, all the four-sensing materials sense CO gas better, but SnO_2_ doped with 0.2% Pd gives better result than pure SnO_2_ as well as SnO_2_ doped with 0.1% or 0.3% Pd. The sensitivity of this 0.2% Pd-doped SnO_2_ sensor is even better than that of the commercial sensor MQ-7, which had shown the best sensitivity in three sensor array. The sensitive material of MQ-7 is also tin oxide that detects CO concentrations in the air with high sensitivity and outputs its reading as an analogue voltage. However, it cannot be used to detect CO in the automobile exhaust, because of its size and other drawbacks. Together with MQ-7, an exhaust gas analyser was also kept inside the gas chamber to determine the level of exhaust gases. The hydrocarbon (130 ppm) and NO_x_ (350 ppm) levels were less, whereas the CO (40,000 ppm) and CO_2_ (110,000 ppm) levels were comparatively higher. On comparing the trend observed in the variation of the exhaust gas levels with that of the sensor values, it resembled the CO levels, thereby suggesting that the voltage change observed must have been caused by CO.

**Figure 9:**
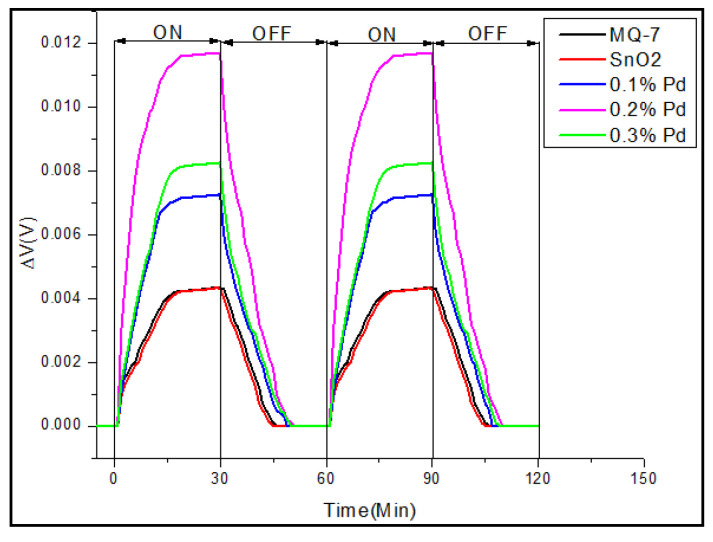
Change in voltage for eight sensor array of pure SnO_2_ as well as with different concentrations 0.1%, 0.2%, 0.3% of palladium and commercial CO sensor MQ-7, during repeatability test @1000 rpm.

Repeatability is the ability of a sensor to repeat a measurement when put back in the same environment and this is very often directly related to accuracy. In order to test the repeatability of the eight sensor array, the engine speed was kept constant at 1000 rpm and the change in voltage was recorded. This result is shown in Figure 9, which proves that the sensor values were reliable for a period of 2 h. An effective strategy to achieve eminent gas-sensing performances is that the sensor should possess high repeatability and long-term stability [24] and in our study, this repeatability is attributed to the unique nanoporous structures of SnO_2_ and the chemical as well as electronic sensitization of palladium.

**Figure 10:**
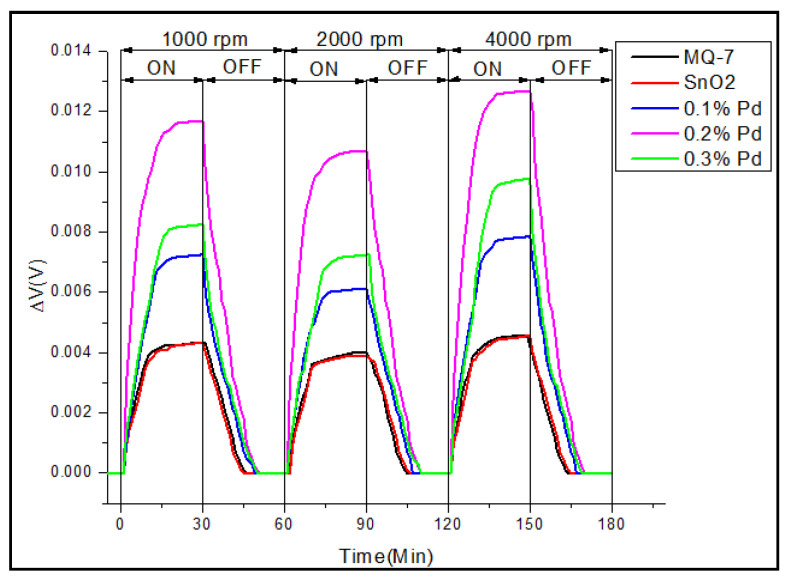
Change in voltage for eight sensor array of pure SnO_2_ as well as with different concentrations 0.1%, 0.2%, 0.3% of palladium and commercial CO sensor MQ-7, @1000 rpm, 2000 and 4000 rpm.

The experiment was repeated for two more different engine speeds 2000 rpm as well as 4000 rpm and the sensitivity was tested. When the amount of oxygen during the combustion is low, CO is emitted as an exhaust product. It has been shown that the CO emission decreases while the speed of the engine rises to nearly 2000 rpm and then is almost constant till 3000 rpm; the CO level again increases reaching maximum at about 4000 rpm and then decreases to a minimum value [52]. Thus in our study, three engine speeds, one lower to 2000 rpm, i.e., 1000 rpm, 2000 rpm, and 4000 rpm are chosen to test the sensitivity of the sensors. The results are shown in Figure 10, which replicate the CO level exhausted from the engine in terms of change in voltage as sensitivity of the sensor. From the data obtained, it is noted that the CO gas coming out of the engine first increases at 1000 rpm, then decreases at 2000 rpm, and again rises at 4000 rpm. This clearly indicates that the real-time testing of the sensors is valid and the sensitivity of the 0.2% Pd-doped SnO_2_ shows maximum sensitivity, or in other words, senses the emission of CO effectively.

Though palladium is expensive, this noble metal is found to be an excellent catalyst for sensing gases like hydrogen, oxides of nitrogen, and hydrocarbons. On optimization of palladium-based hydrogen sensors for sensitivity, it has been shown that, apart from the design of the sensor, the working temperature and the palladium geometry also play vital roles to achieve desired sensitivity [53]. When studies were carried out with Pt-loaded SnO_2_ and Pd-doped SnO_2_, the latter was found to be more selective for hydrogen giving a fast response [54]. The sensitivity and life-time was shown to be higher for Pd-tin reformed microband electrode series, as a nitrate sensor [55]. The sensors using Pd-doped TiO_2_ fibers exhibited encouraging gas sensing characteristics like low operation temperature and adequate gas response [56]. The sensor response to methane was found to be higher and fast-acting, when 1.41% palladium was added to tin dioxide, compared to the non-doped tin oxide [57]. These gases are seen to be sensed by palladium-doped SnO_2_, but at concentrations more than 0.5% palladium [29], which pave chances that only CO gas gets sensed at this lesser concentration of 0.1–0.2% Pd, making this sensor highly selective for CO gas, in that case. It has been shown that 0.2% Pd-loaded SnO_2_ had drastically increased the selectivity of CO against hydrogen than pure tin oxide at 350 °C [58]. However, future studies are carried out to confirm this selectivity of the Pd-doped sensor to carbon monoxide at lower temperature.

Lowering the operation temperature is considered to be an essential criterion in the design of a sensor because though the automobile engine operates at higher temperature, the temperature of the exhaust coming out is comparatively at lower temperature. Hence the sensor that attempts to sense any exhaust gas should function with high specificity at lower temperature and in our study, SnO_2_ with 0.2% Pd showed maximum sensitivity at relatively lower temperature. When the temperature and gas concentration is increased, sensor response for SnO_2_-Pd nanofiber system has also increased [59]. At relatively lower operating temperature, the SnO_2_ nanotubes demonstrate high delicacy and discrimination for acetone detection, when compared with other reported SnO_2_-based sensing materials [60]. SnO_2_ nanofibers, when used as nanosensors in electronic noses, have shown higher sensitivity to NO_2_ when the temperature was low [61]. The temperature-dependent response of PdO showed good sensitivity to various CO concentrations at operating temperatures from 25 °C to 100 °C, because the amount of adsorption of CO varies with temperature [62]. The nanoparticles of PdO/SnO_2_ has a low detection limit and good selectivity to carbon monoxide than other interfering gases, together with low-temperature stability and reversibility, clearly showing that this sensor is a practical low-temperature sensing material for CO [63].

Many attempts have been made to overcome the problems and challenges encountered by the SnO_2_ sensors, such as the effect of interfering gases, the low selectivity and the operation of the sensors with variable temperature profiles. By increasing the surface area of the porous nanocomposites and the spillover effects of palladium nanoparticles, CO sensing characteristics of SnO_2_ had shown to be improved in terms of response, selectivity, and stability, even at low operating temperature [64]. The response of 0.5% palladium-doped graphene-SnO_2_ toward carbon monoxide gas was lower than that of 0.1% Pd [31], however, they have not checked the intermediate concentrations of 0.1%–0.5% Pd. Moreover, there are a few drawbacks in using graphene such as lack of method for large-scale preparation and the necessity of further treatment of graphene to improve its response sensitivity [65]. Earlier, it has been shown that 0.2% Pd-doped SnO_2_ exhibits the best response (80%) toward CO at different operating temperatures within the measurement time [66]. In our study, when SnO_2_ nanoparticles were doped with 0.1%, 0.2%, and 0.3% palladium, the results show an increased sensing capability toward CO gas, the best sensitivity being exhibited by 0.2% Pd-doped SnO_2_ nanoparticles connected as eight sensor array.

## 4. Conclusions

The need of the day to minimize air pollution is a sensor to measure the levels of the major air pollutant, carbon monoxide, which can be specific to CO gas even in the parts-per-million range at lower temperatures. In this attempt, SnO_2_ nanoparticles synthesized from stannous chloride dihydrate chemical compound by chemical precipitation method were doped with 0.1%, 0.2%, and 0.3% palladium, and found to be capable of sensing CO gas among various other gases. The characterization results of the sensing materials were obtained by using XRD and FESEM techniques, which confirms the presence of tin oxide in the final products. XRD indicates the tetragonal structure of the particles and FESEM analysis shows the presence of tin oxide powder with particle size of about 7–20 nm. All the four samples synthesized, viz., pure SnO_2_, SnO_2_ with 0.1% Pd, 0.2% Pd, and 0.3% Pd, were coated as three and eight sensor array, with which the real time testing of the sensor was carried out and the results were compared. On validating, this sensor connected in eight sensor array was able to sense the CO gas with greater efficiency and sensitivity, even higher than the commercially available sensor, among which 0.2% Pd-doped SnO_2_ showed maximum sensitivity to the exhaust CO gas pollutant. However, these that are reported here are preliminary results; further in-depth studies have to be performed on the argument exposed. In future, similar sensor can be designed for sensing other gases by changing the sensing material, and the sensor thus designed can be integrated into a single package.
